# Vibrationally Induced Magnetism in Supramolecular Aggregates

**DOI:** 10.1021/acs.jpclett.3c00157

**Published:** 2023-03-06

**Authors:** J. Fransson

**Affiliations:** Department of Physics and Astronomy, Uppsala University, Box 516, 75120 Uppsala, Sweden

## Abstract

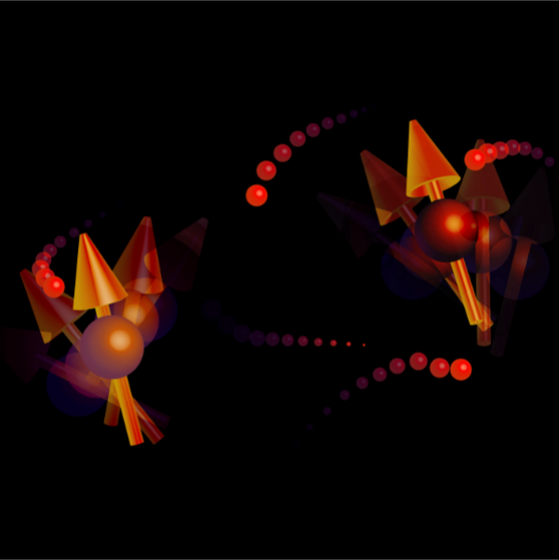

Magnetic phenomena in chemistry and condensed matter physics are considered to be associated with low temperatures. That a magnetic state or order is stable below a critical temperature as well as becoming stronger the lower the temperature is a nearly unquestioned paradigm. It is, therefore, surprising that recent experimental observations made on supramolecular aggregates suggest that, for instance, the magnetic coercivity may increase with an increasing temperature and the chiral-induced spin selectivity effect may be enhanced. Here, a mechanism for vibrationally stabilized magnetism is proposed, and a theoretical model is introduced with which the qualitative aspects of the recent experimental findings can be explained. It is argued that anharmonic vibrations, which become increasingly occupied with an increasing temperature, enable nuclear vibrations to both stabilize and sustain magnetic states. The theoretical proposal, hence, pertains to structures without inversion and/or reflection symmetries, for instance, chiral molecules and crystals.

Magnetism and magnetic phenomena concern central questions in physics and chemistry and remain to be major fields of research. The phenomenology of magnetism pertains to seemingly separated questions of, for instance, room-temperature ferromagnetism,^1−6^ topological matter,^7−10^ and exotic spin excitations (e.g., skyrmions).^11−14^ Furthermore, results from basic science of magnetism have led to important applications in, for example, medicine [e.g., magnetic resonance imaging (MRI)]^15,16^ and data storage (hard drives based on giant magnetoresistance).^17,18^ Recent studies also suggest that magnetic phenomena are not only pertinent in biological contexts but are also crucial for, e.g., oxygen redox reactions, on which aerobic life depends.^19−21^

Ordered magnetic states typically form below a critical temperature, *T*_c_. Above this temperature, higher energy excitations become occupied as a result of thermal fluctuations, which tend to have a randomizing, hence, detrimental effect on the magnetic order. An overwhelming amount of evidence for this behavior has been collected over the years, which naturally has also led to the idea that magnetic order is perceived as a low-temperature collective phase, albeit *T*_c_ may be larger than 1000 K in some compounds.^22,23^ Therefore, from this point of view, the recent reports on supramolecular aggregates that show stable room-temperature ferromagnetism however small if not vanishing magnetic moment at low temperature^24,25^ are indeed surprising. Nevertheless, while the presented measurements suggest an increasing coercive field with the temperature, one would, at least from an entropy argument, still have to expect the existence of a critical temperature above which the magnetic order ceases to exist.

As a destructive mechanism for magnetic order, nuclear vibrations and their collective correspondence, phonons, are considered as a source for decoherence and dissipation of stable magnetic states. It is, therefore, easy to make a connection between increased temperatures and phonon activation, which leads to energy level broadening, occupation of multiple electronic states with competing magnetic properties, and distortions and reformations of nuclear configurations. Hitherto, nuclear vibrations have never been considered as a source for stabilizing magnetic order.

In this letter, a coupling between spin and vibrational degrees of freedom that provides a mechanism for stabilizing finite magnetic moments in molecular aggregates and crystals is proposed. The mechanism is shown to generate an increasing magnetic moment with increasing temperature as well as a critical temperature below which the magnetic moment becomes small or negligible. The source for this property lies in the nature of the vibrational excitations. For sufficiently low temperatures, the nuclear vibrations are dominated by harmonic oscillations around some equilibrium position in which neither charge nor spin polarize the structure. For higher temperatures, anharmonic vibrational modes are occupied, which may cause a net average nuclear displacement from the equilibrium position. Hence, this displacement leads to a local charge polarization, which exerts a force on the local spins. Then, should the compound lack inversion and/or reflection symmetry, a net magnetic moment can be developed and maintained.

Simply described, the effect can be viewed in an dimer of vibrating spins, with spin *S* = ^1^/_2_, which are coupled via indirect exchange through an electronic medium.^26^ Then, while an isotropic spin exchange generates the set of singlet and triplet states, a symmetric anisotropic spin exchange separates the triplet into a singlet and a doublet. Finally, via a spin vibration coupling the nuclear vibrations provides a pseudo-magnetic field, which spin splits the remaining doublet. Regardless of whether the isotropic spin exchange favors a ferromagnetic or antiferromagnetic zero-temperature ground state, the excited states begin to become occupied whenever thermal excitations are available as the temperature rises. Then, the occupation of the spin-split doublet is unequally distributed between the two states, which results in a finite magnetic moment of the dimer.

The question to be addressed here is whether nuclear (ionic and molecular) vibrations can stabilize magnetic configurations or a magnetic order in structures in which the time-reversal symmetry is sustained in the absence of such vibrations. The investigation is stimulated by the experimental observations of room-temperature ferromagnetism in self-assembled ensembles of paramagnetic and diamagnetic supramolecular aggregates or other types of molecular structures.^23−25,27−31^

The discussion is begun by considering the introduced example of the spin dimer **S**_1_ ⊗ **S**_2_, which, albeit simplified, makes it possible to highlight some of the generic aspects of the proposed mechanism. Therefore, the underlying physics can be illustrated by the model Hamiltonian^26^
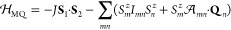
1where **S**_*m*_ and **Q**_*m*_, with *m* = 1 and 2, denote the spin moment and nuclear displacement operator vectors, respectively. The spins are mutually coupled via the isotropic, *J*, and symmetric anisotropic, *I*_*mn*_, spin exchanges as well as coupled to the nuclear displacements through the spin–lattice coupling  (see Figure 1 for an illustration of the comprised interactions). Here, for the sake of clarity, however, without loss of generality, only the *z* projections of the spins have been included in the anisotropic and spin–lattice couplings.

**Figure 1:**
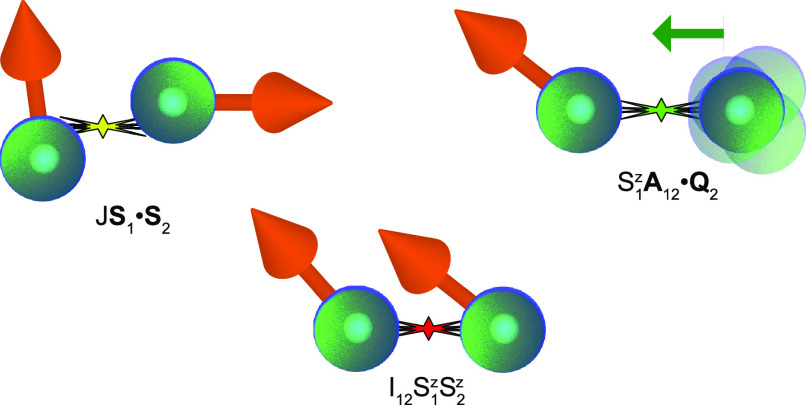
Schematic illustration of the components in the model , describing the (left) isotropic and (middle) symmetric anisotropic spin–spin interaction and (right) spin–lattice interaction.

The spectrum of this model is given by the four energies
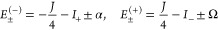
2where the parameters *I*_+_ = *∑*_*mn*_*I*_*mn*_/4, *I*_–_ = *I*_+_ – (*I*_12_ – *I*_21_)/2, , and , in which , whereas ⟨*X*⟩ denotes the expectation value of an operator *X*.

The possible outcomes of this spectrum are conveniently analyzed by first considering all parameters but *J* to vanish. Then, the solutions can be categorized into the singlet (*E*_S_ = 3*J*/4, |*S* = 0, *m*_*z*_ = 0⟩) and triplet (*E*_T_ = −*J*/4, |*S* = 1, *m*_*z*_ = *m*⟩, where *m* = 0 and ±1) states, with a singlet (triplet) ground state whenever the isotropic spin exchange is antiferromagnetic, *J* < 0 (ferromagnetic, *J* > 0). An illustration of this scenario is shown in Figure 2a, for an antiferromagnetic spin exchange.

**Figure 2:**
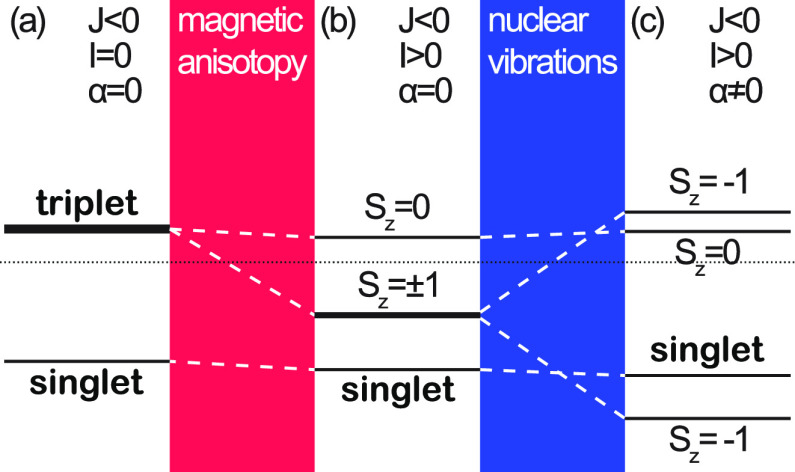
Illustration of a possible energy diagram for the (a) antiferromagnetically coupled spin dimer under the influence of (b) easy axis anisotropy and (c) nuclear vibration.

Starting from the antiferromagnetic ground state, *J* < 0, the introduction of vibrations may result in a strong change of the spin ground state. From the addition of the magnetic and vibrational contributions to the spin excitations, the degeneracies of the states break up and the corresponding energies shift according to the scheme laid out in Figure 2. Structurally, the negative spin exchange *J* leads to the singlet state being the ground state, in the absence of *I*_±_, α, and ζ, whereas the triplet states represent the excited states. While the addition of the magnetic anisotropies, in principle, may lower the energies of the states |*S* = 1, *m*_*z*_ = ±1⟩ below the singlet, it is not likely to happen because the amplitude of the parameters *I*_±_ are often but not always significantly smaller than |*J*|.^26^ In contrast, the energy scales set by α and ζ, which are attributed to the spin–lattice coupling, may assume values with amplitudes well within the order of |*J*| and also larger.^26^ Then, this may lead to a changed ground state from the low-spin (⟨**S**_1_ ⊗ **S**_2_⟩ = 0) to high-spin (⟨**S**_1_ ⊗ **S**_2_⟩ ≠ 0) configuration. The requirements for such a transformation can be formulated in terms of the condition , where . This condition opens up the possibility that it would be sufficient with a small ζ, which occurs whenever . Physically, this means that the spin–lattice couplings  and  are not required to be asymmetric for the nuclear vibrations to sustain the magnetic ground state.

In a more generalized form, one may assume that the localized spin moments as well as the nuclear motion are embedded in an electronic structure, which constitutes the spin–spin and spin–vibration couplings.^26^ In such an environment, it can be shown that the spin–lattice coupling is non-vanishing whenever the background electronic structure has either a viable spin density and/or a spin–orbit coupling. Hence, while the former requirement implies a pre-existing ordered magnetic state of the structure, the latter does not. In the following, it will merely be assumed that the spin–lattice coupling is non-zero, while the specific origin for this state is non-essential. Without assuming a specific form of a general lattice in which the spin and vibrational components are embedded, the considered structure can be summarized in a model of the form
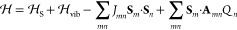
3Here, the first two terms include the local on-site spin (anisotropy) and vibrational (modes and anharmonicity) properties, respectively. The third term describes the spin–spin interactions between the spin moments, whereas the last term accounts for spin–vibration interactions in the lattice. The displacement operator *Q*_*m*_ is expressed in terms of the vibrational creation and destruction operators *a*_*m*_ and *a*_*m*_^†^, respectively, such that *Q*_*m*_ = *a*_*m*_ + *a*_*m*_^†^.

The following investigation is focused on the magnetic moment ⟨**S**_*m*_⟩ per site as well as the collective magnetization ⟨**M**⟩ = *∑*_*m*_⟨**S**_*m*_⟩ in the whole structure. In reciprocal space, the spin operator , where *N* is the number of sites lattice and analogous for the vibration operators *a*_*m*_. The diagonal structure of  is preserved also in reciprocal space under the assumption that *E*_*m*α_ = *E*_α_ for all *m*, which is justified under the assumption that, in the absence of interactions, the local environment of each spin moment is equivalent. It is equally justified to assume that the local vibrational modes are the same for all molecules of the same kind. Therefore, the Hamiltonian for the a single vibrational mode is given by , where the last term contains possible anharmonic properties of the vibrational structure.

The Heisenberg model, –*∑*_*mn*_*J*_*mn*_**S**_*m*_·**S**_*n*_, is simplified by assuming that the exchange parameter is distance-dependent upon the simple form *J*_*mn*_ = *J*(**r**_*m*_ – **r**_*n*_), such that , which is independent of the neighboring sites *n*. Then, the spin–spin interactions can be written as , where **k̅** = −**k**. By the same token, the interaction term is written as , with  and . It should be noticed that **A**_0_ = 0 because there is no net displacement of the compound. The model is in reciprocal space, hence, reformulated to read
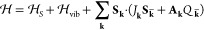
4It should be noticed that this formulation is general and applies equally to orded and disordered compounds, because the derivation does not rely on any assumption about a regular lattice structure.

The conversion of the model into reciprocal space, taking into account the simplifications, leads to the argument applied for the spin dimer above being utilized for each momentum **k**. Indeed, for each **k**, the mean field description, *Q*_**k**_ → ⟨*Q*_**k**_⟩, leads to the pair of spins **S**_**k**_ and , depending upon *J*_**k**_, forming either a singlet or triplet ground state in the limit , whereas the degeneracy of the triplet state breaks for non-vanishing  (cf. Figure 2).

For a spin–lattice coupling , the spectrum of the spin dimer  corresponding to  is for spin ^1^/_2_ given by  and *E*_±_^(+)^(**k**) = −*J*_**k**_/4 ± Ω_**k**_/2, where . The spin moment for the spin dimer, calculated here using , is, therefore, given by
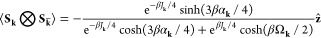
5where 1/β = *k*_B_*T* is the thermal energy in terms of the temperature *T* and the Boltzmann constant *k*_B_. The spin moment is vanishingly small for positive *J*_**k**_ whenever 3|α_**k**_| < *J*_**k**_; that is, the antiferromagnetic regime prevails for sufficiently small spin–lattice coupling. Reversely, the spin dimer acquires a net moment for 3|α_**k**_| > *J*_**k**_ for both ferromagnetic and antiferromagnetic spin exchange *J*_**k**_. Moreover, this net moment changes sign with α_**k**_, as would be expected for a field that is formally similar to a magnetic field.

The **ẑ** -projected spin moment for the spin dimer , where **k** ≠ 0, is plotted in Figure 3 for setups with spin (a) *S* = ^1^/_2_, (b) *S* = 1, (c) *S* = ^3^/_2_, and (d) *S* = 2 and vanishing on-site anisotropy. The plots very well illustrate the vibrationally stabilized magnetic moments of the structures for non-vanishing α_**k**_, whenever the spin exchange *J*_**k**_ < 0. The transition from the zero moment antiferromagnetic state, *J*_**k**_ > 0, to a stabilized moment that is sufficiently strong at the crossover condition 3|α_**k**_| = *J*_**k**_ is conspicuous. These calculations are consistent with the implications that were drawn for the dimer (cf. Figure 2).

**Figure 3:**
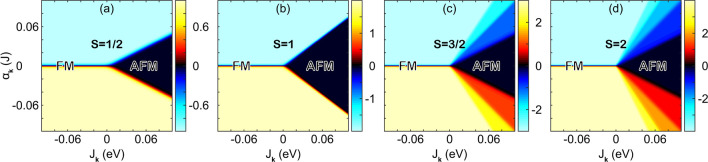
Phase diagrams for spin dimers  with spin (a) *S* = ^1^/_2_, (b) *S* = 1, (c) *S* = ^3^/_2_, and (d) *S* = 2 as a function of the spin exchange *J*_**k**_ and nuclear displacement field . The color scale denotes the total spin moment of the dimer, where black signifies the antiferromagnetic regime, whereas the colored areas correspond to magnetized ferromagnetic regimes. Here, the temperature *T* = 3 K. The ranges for ferromagnetic (FM, *J*_**k**_ < 0) and antiferromagnetic (AFM, *J*_**k**_ > 0) spin exchange are indicated.

The phenomenological basis that the magnetic moment may be stabilized by the nuclear vibrations is thereby set. The next task is, then, to consider a mechanism that can support this phenomenological description.

The above discussion is based on the mean displacement ⟨*Q*_**k**_⟩ being non-vanishing. For simplicity and to study the effect of the mean displacement, consider the linear response expansion, , with respect to a perturbation . To the first non-trivial order in terms of anharmonic processes , the mean displacement is, then, given by
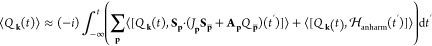
6Then, for a vibrational background that constitutes only harmonic oscillations, that is, , the last contribution in the above expansion vanishes. Under such conditions, the mean displacement and local spin moments are interdependent, such that ⟨*Q*_**k**_⟩ ≠ 0 if and only if ⟨**S**_**k**_⟩ ≠ 0. Hence, harmonic nuclear vibrations may not by themselves lead to a breaking of the time-reversal symmetry. Harmonic nuclear vibrations may, nevertheless, facilitate a strengthening of an already existing magnetic state through a self-consistent buildup of the nuclear displacement field.

In contrast, with the presence of anharmonic vibrations in the structure, time-reversal symmetry may be broken. For instance, using the model , which corresponds to the lowest order anharmonic correction to phonon Hamiltonian, one obtains from the last term in eq 6 the correction
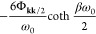
7The temperature dependence of this function is displayed in Figure 4 for vibrational energies ω_0_ ranging between 1 and 200 meV, assuming that Φ_**kk**′_ varies slowly with the temperature. It may literally be seen that the temperature variations are very strong for low energies but wane with increasing energy. This property suggests that slow vibrations, e.g., coherent vibrations that incorporate a larger portion of the structure, can be expected to be crucial for the vibrationally assisted formation of the magnetic order. Reversely, high-frequency vibrations of the nuclei may not contribute to the formation of the magnetic order but may instead be expected to be destructive for the same.

**Figure 4:**
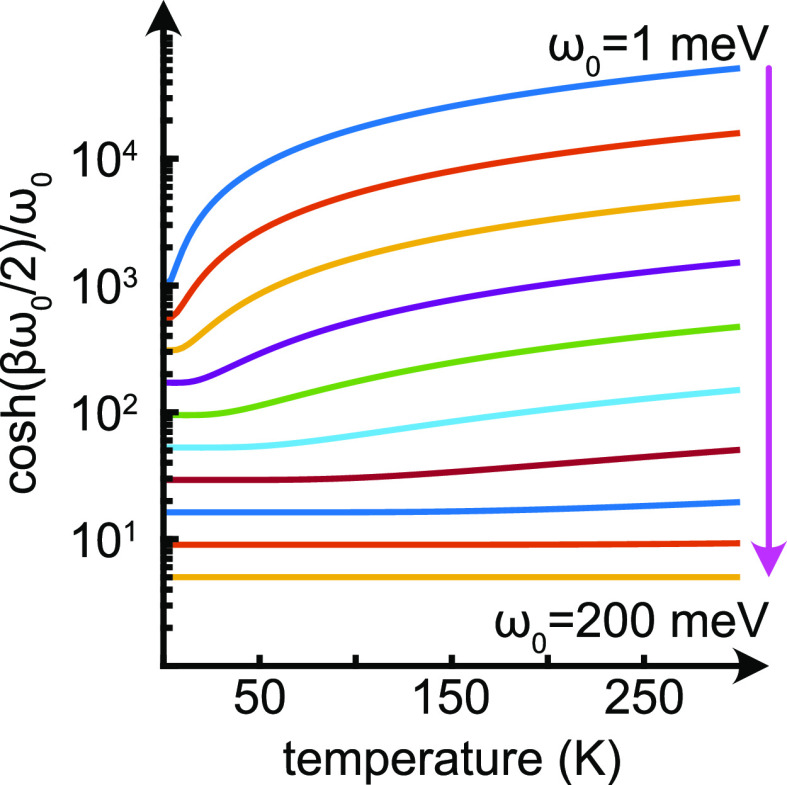
Temperature dependence of coth(βω_0_/2)/ω_0_ for ω_0_ ∈ {1.0, 1.8, 3.2, 5.8, 10.5, 19.0, 34.1, 61.5, 111, 200} meV.

The anharmonic correction does lead to the expected properties for the spin pair , which can be seen in Figure 5. Here, the phase diagrams for the total spin of such a pair with spin *S* = 1 are plotted as a function of the spin exchange *J* and temperature *T*, for the vibrational energies (a) ω_0_ = 0.6 meV, (b) ω_0_ = 1.2 meV, (c) ω_0_ = 1.8 meV, and (d) ω_0_ = 2.4 meV. The four examples provide a good range of the possible magnetic properties that could be expected. For small vibrational energies, for instance, the spin–phonon coupling maintains the ferromagnetic state in a wide temperature range. It, moreover, induces the transition between the antiferromagnetic to ferromagnetic state for a large enough temperature. Then, for increasing vibrational energies, the ferromagnetic state is partially developed throughout the phase diagram but is fully developed at a low temperature in the ferromagnetic regime only. For sufficiently large vibrational energy, finally, the ferromagnetic state is confined to a low temperature in the ferromagnetic regime only.

**Figure 5:**
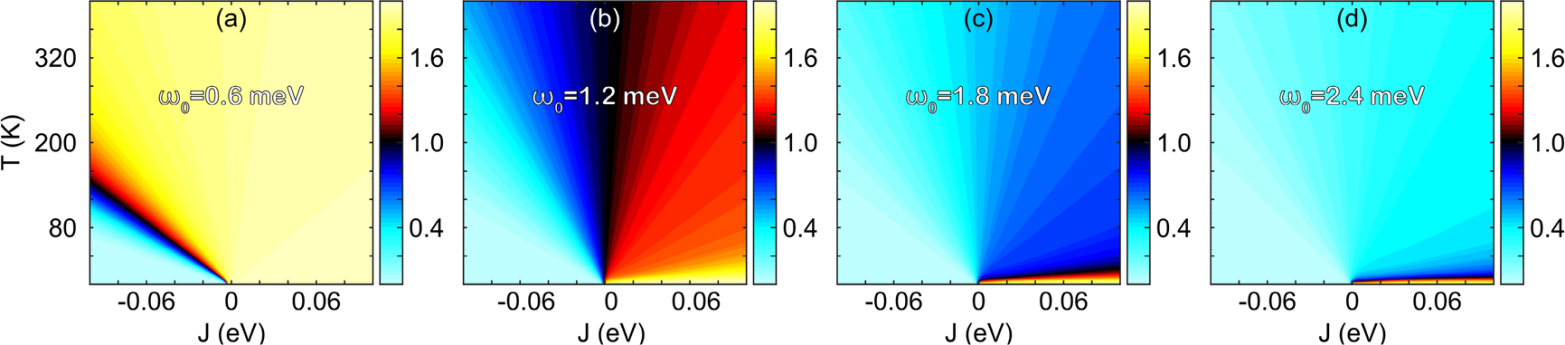
Phase diagrams for spin dimers with spin *S* = 1 and vibrational modes (a) ω_0_ = 0.6 meV, (b) ω_0_ = 1.2 meV, (c) ω_0_ = 1.8 meV, and (d) ω_0_ = 2.4 meV, as a function of the spin exchange *J* and temperature *T*. The color scale denotes the total spin moment of the dimer. Other parameters are *A*_**k**_^(*z*)^Φ = 0.2 μeV.

Finally, to obtain an estimate of the magnetic moment ⟨**S**_*m*_⟩, hence, the magnetization ⟨**M**,⟩ assume, without the loss of generality, a two-dimensional structure, in which the spin-exchange interaction *J*(**r**_*m*_ – **r**_*n*_) can be approximated by a Gaussian distribution function, that is, , where *J* is a constant energy, whereas *r*_S_ is the spatial decay rate. With these assumptions, the **k**-dependent exchange parameter *J*_**k**_ is approximately given by , where *k*_S_ = 2/*r*_S_. Similarly, the spin–lattice coupling **A**_**k**_ can be approximated by , where *k*_A_ = 2/*r*_A_ in terms of the spatial decay rate *r*_A_. Assuming that the coupling parameter Φ_**kk**′_ ≈ Φ varies slowly with the momentum, the spin moment per site is calculated numerically. The plots in Figure 6 illustrate the magnetic moment per site, ⟨**S**_*m*_⟩, in a configuration with (a and c) antiferromagnetic (*J* < 0) and (b and d) ferromagnetic (*J* > 0) spin exchange and spin *S* = ^1^/_2_. Here, an easy plane anisotropy *I* = |*J*| has been introduced to stabilize the antiferromagnetic state at low temperatures for *J* < 0 as well as the corresponding superparamagnetic state for *J* > 0.

**Figure 6:**
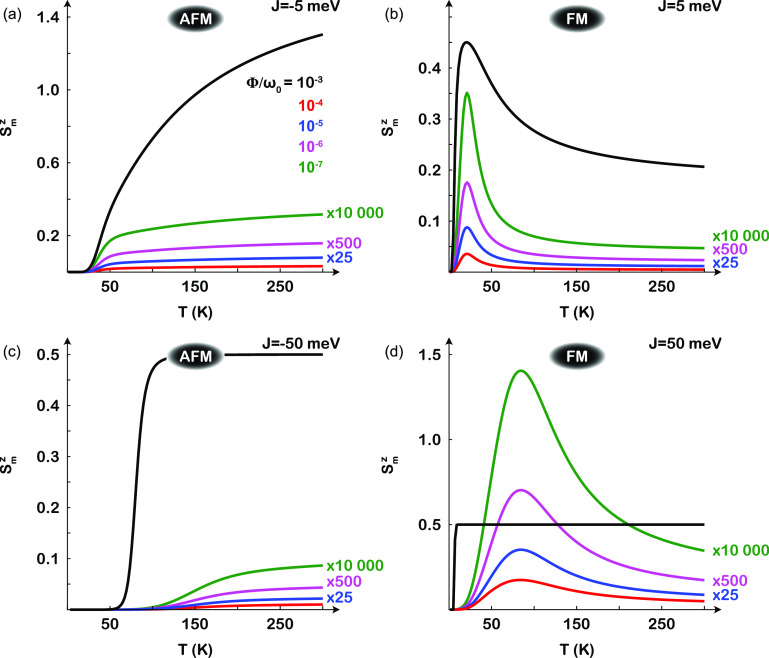
Magnetic moment per site in a configuration with (a and c) antiferromagnetic and (b and d) ferromagnetic spin exchange *J* with spin *S* = 1 as a function of the temperature *T* for a few different strengths of the anharmonic coupling parameter Φ ∈ {10^–7^, 10^–6^, 10^–5^, 10^–4^, 10^–3^}ω_0_, with ω_0_ = 100 μeV. The spin exchange is (a and b) |*J*| = 5 meV and (c and d) |*J*| = 50 meV. Here, *I* = |*J*|, and *r*_S_ = *r*_A_ = 1 Å. The green, magenta, and blue lines are multiplied by 10 000, 500, and 25, respectively, for visibility within the scale.

A conclusion that can be drawn from the plots in Figure 6 is that a spin moment is stabilized under the influence of nuclear vibrations. It can, moreover, be observed that increasing the temperature is not necessarily detrimental for the formation and maintenance of the spin moment. Indeed, in the configuration with antiferromagnetic spin exchange, the moment grows stronger with the temperature throughout the whole range that is considered. The configuration with ferromagnetic spin exchange initially peaks and then decays toward what appears to be a non-zero high-temperature limit. It is also important to notice that it suffices for the anharmonic coupling parameters Φ to be fairly weak, on the order of microelectronvolts, and yet make a significant difference for the magnetic properties. This observation, therefore, suggests that the vibrationally induced magnetism may be present quite abundantly in structures in which inversion and/or reflection symmetries are lacking.

It can be concluded from this discussion that anharmonic vibrations in the structure may lead to a non-vanishing mean displacement, which, in turn, can be the source of stabilization of magnetic moments. However, in a structure with inversion and/or reflection symmetries, the effects of the anharmonicity at different positions would be expected to cancel one another partially or completely, which means that strong observable effects should be sought in compounds that lack such symmetries. In chiral structures, for example, anharmonic contributions would be expected to not cancel out as a result of the intrinsic absence of inversion and reflection symmetries. For instance, anharmonic contributions were proposed to increase the chiral-induced spin selectivity effect with the temperature in azurin.^29^

The results obtained within this letter should be set in perspective with the recent observations of, e.g., increasing coercivity or the enhanced chiral-induced spin selectivity effect with the temperature.^23−25,29−32^ For instance, the molecular crystals investigated in refs (23−25 and 30) show an increasing coercive field up to 300 K but accompanied by decreasing remanent and saturated magnetizations. Such behavior is reproduced by the model for ferromagnetic spin exchange (see Figure 6b). First-principles calculations of the spin exchange indicate that a ferromagnetic order is favored in the compounds investigated in refs (24 and 25), at *T* = 0 K. In the latter article, however, an antiferromagnetic state was reported to be nearly degenerate with the ferromagnetic state. Nuclear vibrations have, moreover, previously been shown to make a significant difference for the theoretical modeling of the chiral-induced spin selectivity effect (see, e.g., refs (29 and 31−35)).

While the proposed theory here is targeted toward supermolecular aggregates, it certainly contains aspects that may also be relevant to explain the magnetic properties found in FeTe compounds^36,37^ and EuSn_2_P_2_.^38^ In the former, a temperature-induced enhancement of the local magnetic moments was attributed to clustering, whereas the critical magnetic temperature was increased by the pressure in the latter, properties that would be addressable in terms of the presented theory.

It has been shown that, by the existence of a coupling between the electronic spin and nuclear vibrations, nuclear vibrations may stabilize and sustain a non-vanishing spin polarization or magnetic moment for a wide range of temperatures. Special focus was paid to compounds comprising localized spin moments, e.g., molecules and molecular aggregates containing transition metal or rare earth elements. The nuclear vibrations generate an effective field acting on the spin moments provided that the vibrations are anharmonic, which is typical for low-symmetry structures, e.g., absence of inversion and/or reflection symmetries. The influence from this induced field increases in strength with the temperature, which, therefore, enables the vibrations to maintain a stable magnetic state also when the spin exchange is too small to provide this. The nature of the spin exchange, whether it favors a ferromagnetic or antiferromagnetic state, becomes irrelevant whenever the temperature is sufficiently large for the vibrationally induced field to dominate the state. It was, finally, argued that the proposed mechanism for stabilizing the magnetic state gives a good qualitative agreement with recent experimental observations of, e.g., the temperature increasing coercivity accompanied by decreasing magnetization. Further experiments that would tie the unusual magnetic properties with, for instance, thermal properties would be desired to facilitate further developments of the proposed theory.

## Supporting Information Available

Transparent Peer Review report available (PDF)
