# Supplementary material for: Vibrationally Induced Magnetism in Supramolecular Aggregates
Source: J Phys Chem Lett. 2023 Mar 6;14(10):2558–64. doi: 10.1021/acs.jpclett.3c00157 (PMC10026173; doi:10.1021/acs.jpclett.3c00157)
Supplement: Supplementary file 1 — jz3c00157_si_001.pdf [file jz3c00157_si_001.pdf]

jz-2023-001578.R1

Name: Peer Review Information for "Vibrationally Induced Magnetism in Supramolecular Aggregates"

First Round of Reviewer Comments

Reviewer: 1

Comments to the Author

The manuscript describes vibrations/phonons induced magnetization in materials that lack inversion or reflection symmetry. The concept broadens the understanding of the temperature effect in magnetic material and provides an understanding of experimental observations. Although the concept was suggested in previous works, also works of the author, this manuscript presents in full the model and provides insight into the mechanism. Hence the work is important and timely. The presentation is clear however readers that are lacking the theoretical background would gain from having a scheme that describes the model.

Another minor issue- the author does not mention in the introduction previous works that discuss various other mechanisms for temperature enhanced magnetism, for example: Phys. Rev. Lett. 107, 216403 (2011) and npj Quantum Mater. 7, 43 (2022).

The manuscript should be published after the author relate to the above comments.

Reviewer: 2

Comments to the Author

The manuscript by J. Fransson is a very nice piece of work and suitable for publication in JPCL. The major advance reported in this manuscript is the physical explanation/physics of magnetic order in supramolecular aggregates/molecular systems at high-temperatures whereby the coercive field was gradually decreasing with lowering the temperature – counterintuitive to our general understanding of ferromagnetism. The first experimental observation of such an astonishing effect was presented in Ref. 24 (JPCL 2016) followed by experimental demonstration in another system in Ref. 25 (ACS Nano 2020); however, the theory was missing till date (for more than 6 years) which has nicely been presented here as “Vibrationally Induced Magnetism”. The theory successfully explains the so called unexpected magnetic properties and much better than broadly assigning thermally-activated ferromagnetism. The inclusion of nuclear vibrations as a source for the magnetic order is really novel and can have immediate significance to explain such anomalous magnetism is other systems.

Author's Response to Peer Review Comments:

**Institutionen för Fysik och Astronomi**
**Dr. Jonas Fransson**
**Professor**

Besöksadress:
Ångströmlaboratoriet,
Polacksbacken
Lägerhyddvägen 5

Postadress:
Box 516
751 21 Uppsala

Telefon:
018 471 5864

Telefax:
018 471 3524

Mobil:
070 167 9264

Hemsida:
http://www.physics.uu.se

Epost:
Jonas.Fransson@physics.uu.se

---

**Department of Physics and Astronomy**
**Jonas Fransson**
**Professor**

Visiting address:
Ångströmlaboratoriet,
Polacksbacken
Lägerhyddvägen 5

Postal address:
Box 516
SE-751 21 Uppsala
SWEDEN

Telephone:
+46 18 471 5864

Telefax:
+46 18 471 3524

Cell:
+46 70 167 9264

Web page:
http://www.physics.uu.se

Email:
Jonas.Fransson@physics.uu.se

Dear Professor Editor,

I herewith submit my manuscript *Vibrationally Induced Magnetism in Supramolecular Aggregates*, by J. Fransson, for consideration for publication in the Journal of Physical Chemistry Letters.

I am truly thankful for the positive reviews of the manuscript, only suggesting the addition of a schematic figure of the proposed model and adding two references. These additions have been made to the manuscript, where I saw it fit to cite also the paper [*Phys. Rev. B*, **2011**, *84*, 045124].

With this, quite minor, revision requested, I hope that the paper meets the criteria set by the Journal of Physical Chemistry Letters.

Yours sincerely,

Jonas Fransson
